# On the systematics of *Phlebotomus betisi* and two new related species from Laos with proposal of the new subgenus *Lewisius*[Fn FN1]

**DOI:** 10.1051/parasite/2023021

**Published:** 2023-06-09

**Authors:** Khamsing Vongphayloth, Fano José Randrianambinintsoa, Khaithong Lakeomany, Nothasine Phommavanh, Nalia Mekarnia, Mohd Shahar Khadri, Matthieu L. Kaltenbach, Antoine Huguenin, Jean-Philippe Martinet, Jérôme Depaquit

**Affiliations:** 1 Institut Pasteur du Laos, Laboratory of Vector-Borne Diseases, Samsenhai Road, Ban Kao-Gnot, Sisattanak District 3560 Vientiane Lao PDR; 2 Faculté de Pharmacie, Université de Reims Champagne-Ardenne, SFR Cap Santé, EA7510 ESCAPE-USC ANSES VECPAR Reims France; 3 Ministry of Health Kuala Lumpur Malaysia; 4 Centre Hospitalo-Universitaire, pôle de Biologie territoriale, Laboratoire de Parasitologie-Mycologie 51092 Reims France; 5 URE Dengue et Arboviroses, Pasteur Network, Institut Pasteur de Nouvelle Calédonie Nouméa Nouvelle Calédonie

**Keywords:** Phlebotomine sandflies, Laos, *Phlebotomus*, New subgenus, New species, Systematics

## Abstract

*Phlebotomus betisi* was described from Malaysia and classified after its description in the subgenus *Larroussius*. It was the only species to have a pharyngeal armature composed of dot-like teeth and an annealed spermatheca whose head is carried by a neck in females. Males were characterized by having a style bearing five spines and a simple paramere. The study of sandflies originating from a cave in Laos enabled us to discover and describe two sympatric species close to *Ph. betisi* Lewis & Wharton, 1963 and new for Science: *Ph. breyi* Vongphayloth & Depaquit n. sp., and *Ph. sinxayarami* Vongphayloth & Depaquit n. sp. They were characterized morphologically, morphometrically, geomorphometrically, molecularly, and proteomically (MALDI-TOF). All approaches converged to validate the individualization of these species whose morphological differential characters lay in the two genders by the observation of the interocular suture and by the length of the last two segments of the maxillary palps. In males, the length of the genital filaments discriminates these species. Females are distinguished by the length of the ducts of the spermathecae as well as by the narrow or enlarged shape of the neck bearing their head. Lastly, the particular position of the spines of the gonostyle coupled with molecular phylogeny led us to remove these three species from the subgenus *Larroussius* Nizulescu, 1931 and to classify them in a new subgenus: *Lewisius* Depaquit & Vongphayloth n. subg.

## Abstract (fr)

*Phlebotomus betisi* a été décrit de Malaisie et fut classé après sa description dans le sous-genre *Larroussius*. C’était la seule espèce à posséder chez la femelle une armature pharyngienne composée de dents en forme de points et à avoir une spermathèque annelée dont la tête est portée par un cou. Les mâles se caractérisaient par un style porteur de cinq épines et par un paramère simple. L’étude de Phlébotomes originaires d’une grotte du Laos nous a permis de découvrir et de décrire deux espèces sympatriques proches de *Ph. betisi* Lewis & Wharton, 1963 et nouvelles pour la Science : *Ph. breyi* Vongphayloth & Depaquit n. sp., et *Ph. sinxayarami* Vongphayloth & Depaquit n. sp. Elles ont été caractérisées morphologiquement, morphométriquement, géomorphométriquement, moléculairement et protéomiquement (MALDI-TOF). Toutes ces approches convergent pour valider l’individualisation de chacune de ces espèces dont les caractères morphologiques différentiels reposent dans les deux sexes par l’observation de la suture interoculaire et par la longueur des deux derniers segments des palpes maxillaires. Chez les mâles, la longueur des filaments génitaux discrimine ces espèces. Les femelles sont distinguées par la longueur des conduits des spermathèques ainsi que par la forme étroite ou élargie du cou portant la tête de ces spermathèques. Enfin, la position particulière des épines sur le gonostyle couplée à une phylogénie moléculaire nous amène à extraire ces trois espèces du sous genre *Larroussius* Nitzulescu, 1931 pour les classer dans un nouveau sous-genre : *Lewisius* Depaquit & Vongphayloth n. subg.

## 1 Introduction

Phlebotomine sandflies represent a tiny blood sucking insect group which has been proven to be the vector of several human pathogens for decades [2, 3]. In the Old World, sandflies belong to eight genera including *Phlebotomus* Rondani & Berté, 1840; *Sergentomyia* França & Parrot, 1919; *Spelaeophlebotomus* Theodor, 1948; *Spelaeomyia* Theodor, 1948; *Idiophlebotomus* Quate & Fairchild, 1961; *Parvidens* Theodor & Mesghali, 1964; *Grassomyia* Theodor, 1958 and *Chinius* Leng, 1987 [43].

Genus *Phlebotomus* is currently divided into 14 subgenera including *Phlebotomus* Rondani & Berté, 1840; *Adlerius* Nitzulescu, 1931; *Larroussius* Nitzulescu, 1931; *Anaphlebotomus* Theodor, 1948; *Australophlebotomus* Theodor, 1948; *Euphlebotomus* Theodor, 1948; *Paraphlebotomus* Theodor, 1948; *Synphlebotomus* Theodor, 1948; *Kasaulius* Lewis, 1982; *Abonnencius* Morillas Márquez, Castillo Remiro & Ubeda Ontiveros, 1984; *Transphlebotomus* Artemiev & Neronov, 1984; *Legeromyia* Rahola, Depaquit & Paupy, 2013; *Madaphlebotomus* Depaquit, Léger & Randrianambinintsoa, 2015 and *Artemievus* Depaquit, 2022 [9, 12, 14, 27, 38, 43, 46, 47].

The subgenus *Larroussius* was created by Nitzulescu in 1931 [30] using *Ph. major* Annandale, 1910 [4] as the type species. Based on morphological characters, the *Larroussius* subgenus was originally described as follows: (i) cibarium without armature, (ii) spermathecae segmented with a long (variable) terminal process (neck) carrying the terminal knob, and (iii) pharynx similar to that of *Ph. major*; Theodor, 1948 [46] proposed: (i) short style with five long spines with two terminal spines and three spines near the middle of segment, (ii) simple parameres with a club-shaped apex, (iii) long parameral sheath (aedeagus) of variable shape, (iv) pharynx with armature of numerous small point-like teeth, and (v) spermathecae segmented with a long terminal process.

In Southeast Asia (SE-Asia), the only known species classified in the subgenus *Larroussius* is *Ph. betisi* Lewis & Wharton, 1963 [29]. This species was described from eight females caught in a cave in Betis, Gua Musang (Malaysia). These authors noted that *Ph. betisi* spermathecae look like those of *Ph. major* (i.e., close to those of *Larroussius*) but noticed that the bead-like segments and the narrowness of the process (meaning the terminal knob (head) carried by a neck) are unusual and that the systematic position of *Ph. betisi* could be clarified by the discovery of a male specimen. In 1978, Lewis [28] classified *Ph. betisi* in the subgenus *Larroussius*, a position adopted in papers published later by Lewis, 1982 [27]; Artemiev & Neronov, 1984 [9]; and by Seccombe et al., 1993 [43]. In 2008, the male of *Ph. betisi* was described by Khadri et al. [25] from specimens caught in Kota Gelanggi cave of Pahang, 20 km away from the type-locality. In this description, the authors indicated that *Ph. betisi* male specimens could be classified in the subgenus *Larroussius* by the morphology of the genitalia (meaning a gonostyle with 5 spines, a simple paramere without club-shaped apex or without a ventral tubercle, and the lack of basal process on the gonocoxite). The position of spines on the style, however, was different from that of other *Larroussius* specimens.

In the present paper, we describe two species new to science from Laos, closely related to *Ph. betisi*, and propose to create a new subgenus to include these three species.

## 2 Methods

### 2.1 Sandfly collection

**Figure 1:**
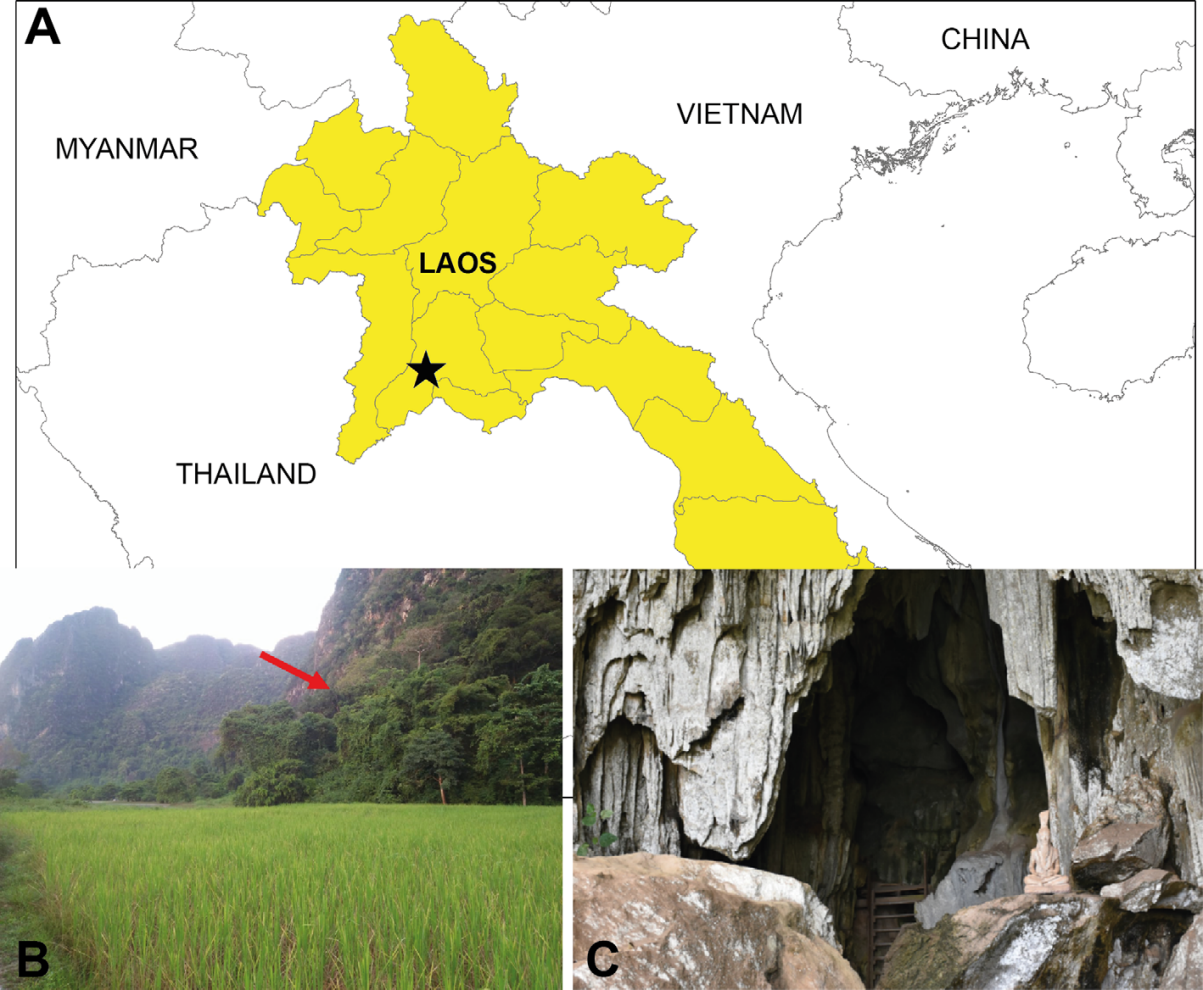
A: the star indicates the location of Pha Nok Kok cave in Feung district, Vientiane province, Laos; B: Pha Nok Kok cave location in the hills (red arrow); C: Pha Nok Kok cave entrance where the sandflies were caught.

Sandflies were collected between May 11 and May 17, 2019 from karstic limestone near the entrance of Pha Nok Kok cave in Feung district, Vientiane province, Laos (locality: 18°30′ N, 101°59′ E, Fig. 1). Sandflies were collected overnight (from 5:00 pm to 6:00 am) using standard CDC light traps (John W. Hock Company, Gainesville, FL, USA). Sandfly samples were then sorted and stored in 95% ethanol. An aliquot was stored dry in silica gel at –20 °C then transferred to the laboratory for further analysis.

### 2.2 Sample processing and morphological analysis

Specimens stored in 95% ethanol were mounted:

- *in toto* for morphological analysis: head, thorax, wings and genitalia were cut-off in a drop of ethanol. Soft tissues were lysed in a bath of 10% KOH then bleached in Marc-André solution, and mounted between microscope slide and cover slide in Euparal^®^ for species identification after dehydration in successive alcoholic baths.
- Partially for molecular studies: head, wings and genitalia were cut-off and mounted directly in Euparal^®^ as described above. Thorax and abdomen were transferred to an Eppendorf 1.5 mL tube with the same labelling as the slide and stored at –20 °C until analysis. All engorged females were processed according to this protocol.

For MALDI-TOF, dry specimens stored at –20 °C were prepared as follows: head, wings and genitalia were cut-off and mounted directly in either Macroinvertebrate Mounting Medium (Polysciences, Inc. Warrington, PA, USA) or in Euparal^®^ as described above. Thorax and abdomen were transferred separately to two Eppendorf 1.5 mL tubes with the same labelling as that of the slide and stored at –20 °C until MALDI-TOF (using the thorax) and molecular analysis (using the abdomen).

The mounting slides were observed on an Olympus BX50 microscope coupled with a DP 26 Olympus camera. Measurements and counting of several characters were performed by using Stream Essentials software (Olympus, Tokyo, Japan), as previously explained [11]. Drawings were made using the *camera lucida* installed on the microscope.

### 2.3 Molecular analysis

#### 2.3.1 Cytochrome b gene (cyt b)

The abdomen of the specimen stored in a 1.5 mL vial was processed by adding 0.5 mL of 1× Phosphate Buffered Saline (PBS) and Lysing Matrix E zirconium beads (MP Biomedicals, Santa Ana, CA, USA) and homogenized for 10 min at a vibration frequency of 25/s in a TissueLyser II system (QIAGEN, Hilden, Germany). After grinding, beads and tissues were spun down by centrifugation for 5 min at 3000 rpm. To obtain total nucleic acid, 100 μL of each sample were extracted and purified using a NucleoSpin^®^8 extraction kit, following the manufacturer’s protocol and using an elution volume of 100 μL. All polymerase chain reaction (PCR) amplifications were carried out in a 50 μL volume containing 5 μL of extracted DNA and 45 μL PCR Master Mix (Promega, Madison, WI, USA), containing 50 pmol of each primer targeting cyt b: C3B-PDR (5′–CAYATTCAACCWGAATGATA–3′) and N1N-PDR (5′–GGTAYWTTGCCTCGAWTTCGWTATGA–3′), according to previously published conditions [17]. Sequencing reactions were performed using a BigDye Terminator v1.1 cycle sequencing kit (Applied Biosystems, Waltham, MA, USA). Sequence chromatograms from both strands were obtained on an automated sequence analyzer ABI3500XL (Applied Biosystems).

Phylogenetic analysis was based on aligned sequences. The maximum likelihood (ML) tree was constructed by MEGA 11 [45] using the substitution models selected by Model test [36] with an Akaike information criterion (AIC) of HKY85 [21]. We have included five specimens of *Ph. breyi* n. sp. and five specimens of *Ph. sinxayarami* n. sp. as well as two Malaysian specimens of the closely related *Ph. betisi* and 53 specimens representing 53 species of the genus *Phlebotomus*, as indicated in Table 1. *Idiophlebotomus longiforcep*s was selected to serve as an outgroup.

**Table 1:** Specimens used for the molecular study.

Genus	Subgenus	Species	Origin	GenBank accession number
*Phlebotomus*	*Larroussius*	*Ph. longicuspis*	Burkina Faso	AY700012
		*Ph. perfiliewi*	Algeria	KF680820
		*Ph. galilaeus*	Cyprus	KF680828
		*Ph. transcaucasicus*	Iran	KF680835
		*Ph. ariasi*	France	HM131112
		*Ph. chadlii*	Algeria	HM131080
		*Ph. orientalis*	Sudan	KU559573
		*Ph. perniciosus*	Tunisia	MW305409
		*Ph. keshishiani*	Afghanistan	HQ204193
		*Ph. tobbi*	Cyprus	OL376918
		*Ph. neglectus*	Greece	OL376973
		*Ph. syriacus*	Israel	KC329644
		*Ph. langeroni*	Spain	LT223559
	*Lewisius* subg. nov.	*Ph. betisi*	Malaysia	OQ784674
		*Ph. betisi*	Malaysia	OQ784675
		*Ph. breyi* n. sp. male	Laos	OQ784676
		*Ph. breyi* n. sp. male	Laos	OQ784677
		*Ph. breyi* n. sp. male	Laos	OQ784678
		*Ph. breyi* n. sp. female	Laos	OQ784679
		*Ph. breyi* n. sp. female	Laos	OQ784680
		*Ph. sinxayarami* n. sp. male	Laos	OQ784681
		*Ph. sinxayarami* n. sp. female	Laos	OQ784682
		*Ph. sinxayarami* n. sp. male	Laos	OQ784683
		*Ph. sinxayarami* n. sp. male	Laos	OQ784684
		*Ph. sinxayarami* n. sp. female	Laos	OQ784685
	*Adlerius*	*Ph. comatus*	Iran	JX885988
		*Ph. longiductus*	Iran	JX885993
		*Ph. kabulensis*	Iran	JX885994
		*Ph. halepensis*	Iran	JX885995
		*Ph. brevis*	Iran	JX885998
		*Ph. arabicus*	Israel	KC329634
		*Ph. creticus*	Greece	MT501636
		*Ph. balcanicus*	Iran	MT501639
		*Ph. chinensis*	China	HM747234
		*Ph. turanicus*	Afghanistan	HM803195
		*Ph. simici*	Greece	MT552618
	*Transphlebotomus*	*Ph. mascittii*	Slovenia	MG800324
		*Ph. canaaniticus*	Israel	KC329646
		*Ph. simonahalepae*	Romania	MZ647524
		*Ph. killicki*	Greece	OL376894
	*Anaphlebotomus*	*Ph. stantoni*	Vietnam	KM409498
		*Ph. rodhaini*	Senegal	KM409501
	*Euphlebotomus*	*Ph. argentipes*	India	KM409508
		*Ph. kiangsuensis*	Thailand	OQ784686
		*Ph. mascomai*	Thailand	OQ784687
		*Ph. barguesae*	Thailand	KM409509
	*Madaphlebotomus*	*Ph. berentiensis*	Madagascar	KM409502
		*Ph. fontenillei*	Madagascar	KM409504
		*Ph. vaomalalae*	Madagascar	JX512360
		*Ph. vincenti*	Madagascar	KM409505
		*Ph. artemievi*	Madagascar	MN346688
		*Ph. fertei*	Madagascar	KM409506
	*Phlebotomus*	*Ph. bergeroti*	Algeria	KJ480973
		*Ph. papatasi*	Jordan	KY990733
		*Ph. duboscqi*	Cameroon	MH577174
	*Paraphlebotomus*	*Ph. sergenti*	Iran	DQ840405
		*Ph. caucasicus*	Iran	EF017364
		*Ph. riouxi*	Tunisia	EU935827
		*Ph. chabaudi*	Tunisia	EU935814
		*Ph. jacusieli*	Israel	KC329638
		*Ph. saevus*	Israel	KC329640
		*Ph. similis*	Greece	OL376910
		*Ph. kazeruni*	Israel	KC329635
	*Artemievus*	*Ph. alexandri*	Algeria	KJ480981
	*Synphlebotomus*	*Ph. saltiae*	Israel	KF483677
*Idiophlebotomus*		*Id. longiforceps*	Thailand	KT878756

#### 2.3.2 Blood meal analysis

The abdomen of each engorged female was processed individually. Total DNA was extracted with a QIAamp DNA mini kit (QIAGEN GmbH), according to the manufacturer’s recommendations. The extracted DNA was eluted in a final volume of 100 μL of AE buffer. For each extraction run, we included a positive control.

To check the bloodmeal origin of engorged females, we amplified the prepronociceptin (PNOC) gene using PNOC-F (forward): 5′–GCATCCTTGAGTGTGAAGAGAA–3′ and PNOC-R (reverse): 5′–TGCCTCATAAACTCACTGAACC–3′ primers, according to the conditions described in the literature [20]. Amplicons were analyzed by electrophoresis in 1.5% agarose gel containing gelgreen. Direct sequencing in both directions was performed with the primers used for DNA amplification.

### 2.4 MALDI-TOF MS Analysis

Matrix Associated Laser Desorption-Ionization – Time Of Flight mass spectrometry (MALDI-TOF) was performed as already described [22]. Briefly, thoraxes, including legs of the specimens, were placed in formic acid (10 μL; Sigma-Aldrich, Lyon, France) and manually ground with a Teflon pestle. Acetonitrile (10 μL Sigma-Aldrich) was then added and after a brief centrifugation step (2 min at 10,000 rpm), 1 μL of supernatant was spotted on a 96-well steel MALDI target plate (Bruker Daltonics, Champs-sur-Marne, France). Specimens were deposited in quadruplets. After complete drying, 1 μL of HCCA matrix solution (Bruker Daltonics) was added and the plates dried at room temperature. Spectra were then acquired using a Bruker Microflex LT MALDI–TOF spectrometer. For each well, spectra acquisition was repeated at least eight times. The Bacterial Test Standard provided by Bruker Daltonics was used for instrument calibration. Lastly, spectra were visually checked using FlexAnalysis v3.4 and imported in Biotyper Compass Explorer v4.1.100 for analysis.

The Bruker “MALDI Biotyper Preprocessing Standard Method” was used for the creation of main spectra profiles (MSP). High-quality spectra were selected for incrementing the local *Ph. Larroussius* database (at least 10 good quality spectra by MSP). Hierarchical cluster analysis (HCA) was performed using both the Euclidean and correlation method and the Ward algorithm for clustering with the MSP dendrogram tool of Compass Explorer.

Primary Component Analysis (PCA) was performed using custom R scripts based on the MALDIQuant, MALDIrppa and FactoMineR packages [19, 26, 31].

### 2.5 Geometric morphometric analysis

Geometric morphometric analysis [37] was carried out on 50 female sandfly wings (*Ph. sinxayarami n* = 39 and *Ph. breyi n* = 11) and 19 male sandfly wings (*Ph. sinxayarami n* = 10 and *Ph. breyi n* = 9). Briefly, fuchsin-stained wings were photographed under ×100 magnification. Work files were then built with TPS Util^©^ version 1.76, and 16 landmarks (LM) were digitized with TPSDig^©^ version 1.40 [42]. Coordinates of scaled 16 landmarks were imported in R software Version 1.4.1103 [41] and processed with the geomorph package [1, 10]. Coordinates were aligned by performing a procrustes superimposition and centroid sizes (CS) were computed. Disparity between CS from the different novel species and between sexes was tested by means of a Wilcoxon test with *p-*values < 0.05 considered significant. Main shape LM disposition from male (*n* = 8) and female (*n* = 3) *Ph. betisi* was computed to serve as a reference for comparison with *Ph. breyi* and *Ph. sinxayarami*. Deformation grids were plotted to identify the main differences between LM positions. Principal Component Analysis (PCA) was performed on *Ph. breyi* and *Ph. sinxayarami* to assess whether morphologic differences could be used to separate these species. Plots were generated with R packages geomorph, factominer, factoextra and ggplot2 [26, 50].

## 3 Results

### 3.1 Molecular analysis

#### 3.1.1 Cytochrome b gene

**Figure 2:**
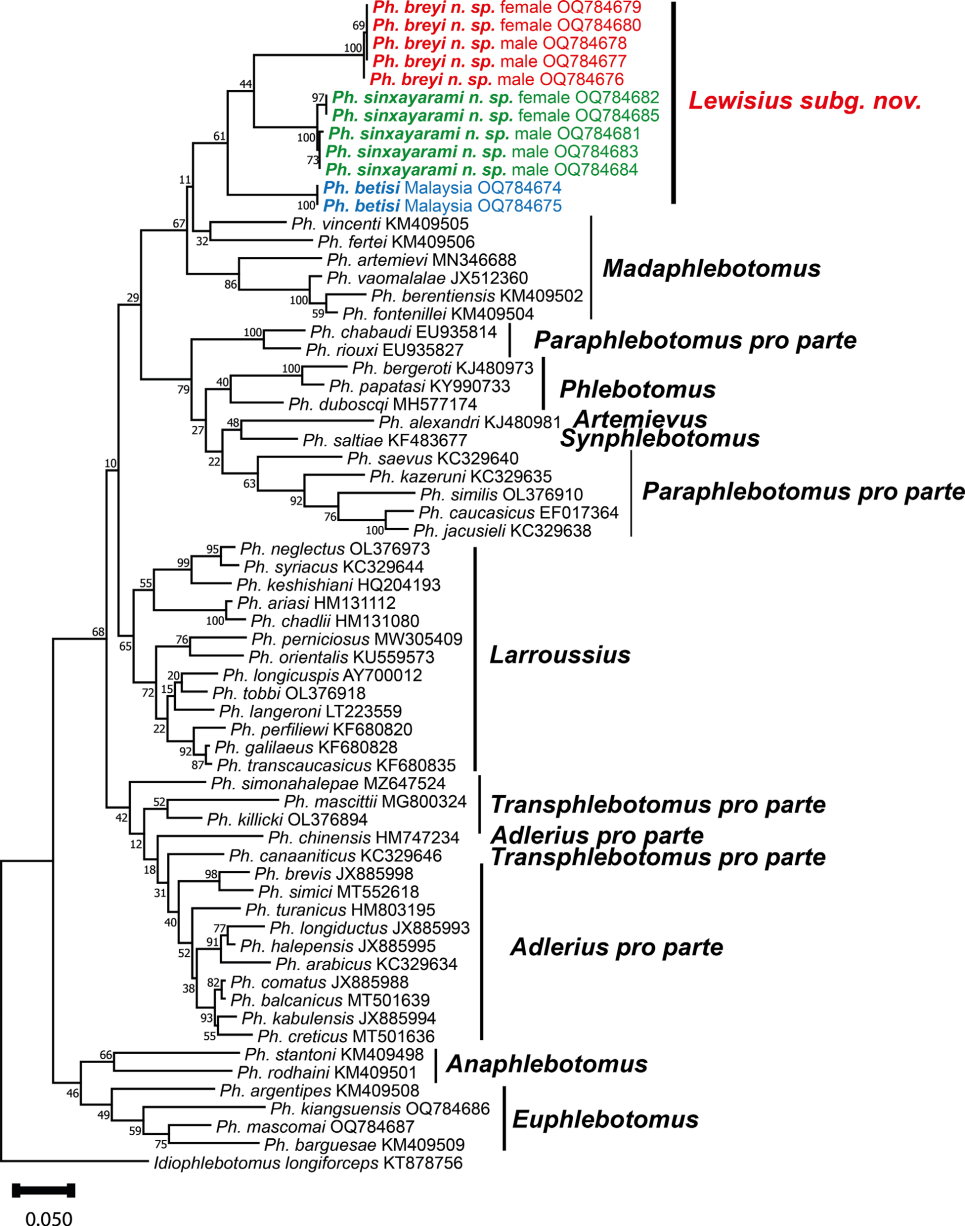
Maximum likelihood phylogenetic tree of partial cyt b gene of the species within *Lewisius* n. subg. and *Larroussius* subgenus. Numbers on branches estimated by 100 replication bootstrap support. Species within *Lewisius* are separated from those within *Larroussius* with a bootstrap value of 100%.

The 405 bp database included 223 variable sites and 182 informative sites for parsimony. A maximum likelihood tree based on this database is shown in Figure 2. The estimation of evolutionary divergence over sequence pairs between and within subgenera is provided in Table 2 and between and within the species *Ph. betisi*, *Ph. breyi* n. sp. and *Ph. sinxayarami* n. sp. in Table 3.

**Table 2:** Number of base differences per site from averaging over all sequence pairs between subgenera and genera (thin style) and within subgenera (bold style) calculated using a *p*-distance model on the cyt b dataset.

	*Lewisius*	*Larroussius*	*Adlerius*	*Transphlebotomus*	*Anaphlebotomus*	*Euphlebotomus*	*Madaphlebotomus*	*Paraphlebotomus*	*Artemievus*	*Synphlebotomus*	*Phlebotomus*	*Idiophlebotomus*
*Lewisius* subg. nov.	**0.102**											
*Larroussius*	0.185	**0.093**										
*Adlerius*	0.180	0.133	**0.074**									
*Transphlebotomus*	0.194	0.147	0.127	**0.122**								
*Anaphlebotomus*	0.222	0.196	0.187	0.200	**0.179**							
*Euphlebotomus*	0.219	0.190	0.186	0.196	0.184	**0.146**						
*Madaphlebotomus*	0.175	0.173	0.177	0.196	0.215	0.211	**0.128**					
*Paraphlebotomus*	0.203	0.181	0.162	0.199	0.222	0.219	0.213	**0.139**				
*Artemievus*	0.231	0.204	0.184	0.204	0.246	0.227	0.233	0.205	**n/a**			
*Synphlebotomus*	0.176	0.171	0.146	0.186	0.206	0.197	0.202	0.164	0.157	**n/a**		
*Phlebotomus*	0.201	0.181	0.158	0.174	0.222	0.216	0.210	0.192	0.188	0.142	**0.126**	
*Idiophlebotomus*	0.224	0.217	0.222	0.237	0.242	0.215	0.230	0.257	0.258	0.230	0.250	**n/a**

**Table 3:** Number of base differences per site from averaging over all sequence pairs between *Lewisius* subg. nov. species (thin style) and within *Lewisius* subg. nov. species (bold style) calculated using a *p*-distance model on the cyt b dataset.

	*Ph. betisi*	*Ph. breyi* n. sp.	*Ph. sinxayarami* n. sp.
*Ph. betisi*	**0**		
*Ph. breyi* n. sp.	0.145	**0.001**	
*Ph. sinxayarami* n. sp.	0.140	0.152	**0.008**

The maximum likelihood phylogenetic tree of cyt b showed that *Ph. betisi* from Malaysia, *Ph. breyi* and *Ph. sinxayarami* are grouped together. The subgenus *Madaphlebotomus*, although appearing as paraphyletic, is a sister-group of these species and is separated from subgenus *Larroussius* with strong maximum statistical support (bootstrap = 100%) (Fig. 2). This result confirms our observations on morphologic characters of *Lewisius* n. subg. (as described below). Phylogenetic analysis also supports our morphologic association of male and female. *Phlebotomus breyi* of both sexes have long palps and incomplete interocular sutures. Females have long spermathecal ducts and males have long aedeagal ducts. Both sexes of *Ph. sinxayarami* have shorter palps, especially p4, and complete interocular sutures. Females have shorter spermathecal ducts and males have shorter aedeagal ducts than those of *Ph. breyi* (see details in description below).

The genetic distances between and within subgenera and species are listed in Table 2 and Table 3, respectively and highlight important differences.

#### 3.1.2 Bloodmeal analysis

The blood meal of one engorged *Ph. sinxayarami* n. sp. female was successfully identified. The sequence homology was >99% with several sequences (including GenBank accession number XM_044939717.2) of water buffalo (*Bubalus bubalis*) that were observed around the entrance of Pha Nok Kok cave.

### 3.2 Wing morphometrics

**Figure 3:**
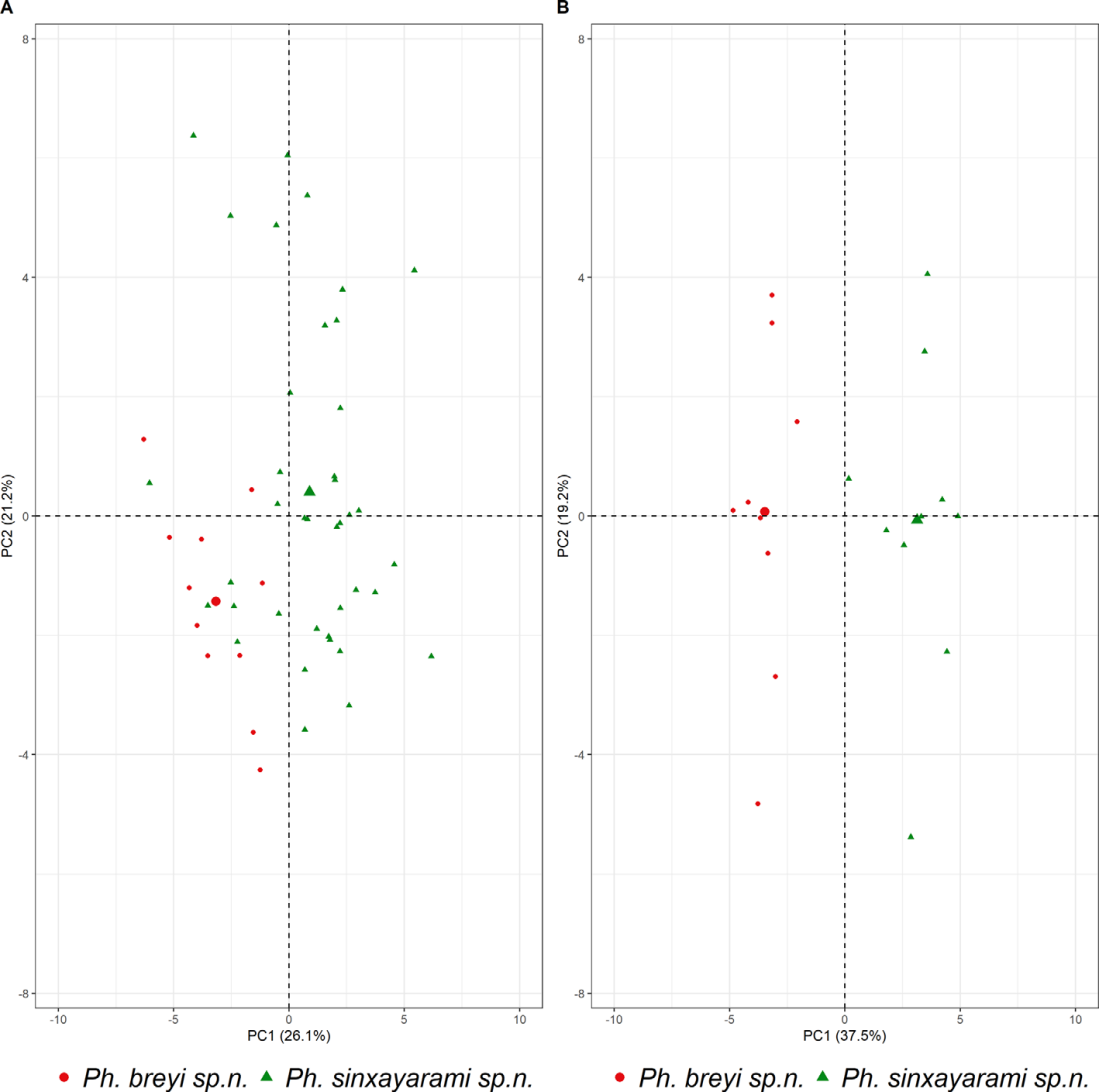
Morphometric analysis. Principal Component Analysis of wings landmarks of *Phlebotomus* (*Lewisius*) *breyi* n. sp. and *Phlebotomus* (*Lew.*) *sinxayarami* n. sp. for females (A, on the left) and males (B, on the right). Biplot of the first two principal components (PC1 and PC2).

**Figure S1:**
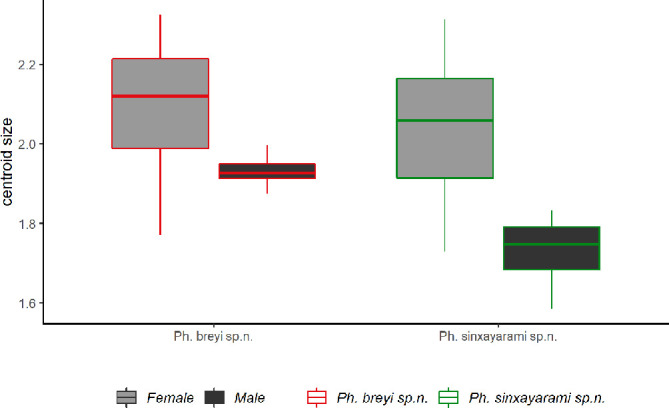
Boxplot of centroid sizes of wings landmarks of *Phlebotomus* (*Lewisius*) *breyi* n. sp. and *Phlebotomus* (*Lewisius*) *sinxayarami* n. sp. for male and female specimens.

**Figure S2:**
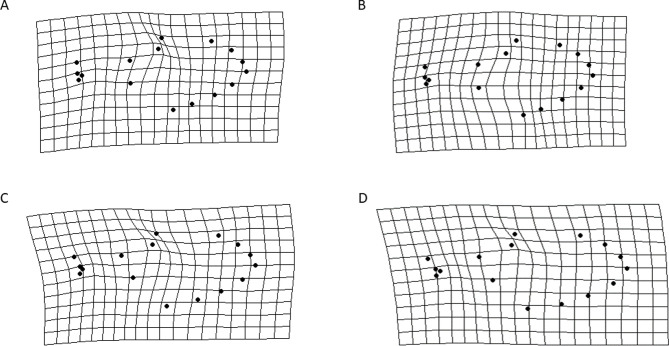
Deformation grids of wings landmarks of *Phlebotomus* (*Lewisius*) *breyi* n. sp. and *Phlebotomus* (*Lewisius*) *breyi* n. sp. for males and females using *Ph. betisi* as reference.

Centroid sizes of *Ph. breyi* and *Ph. sinxayarami* are represented in Figure S1. The Wilcoxon test performed on CS showed no differences between the size of females from *Ph. breyi* and *Ph. sinxayarami* (*p*-value = 0.1622). In contrast, a significant difference between the two male populations was found (*p*-value = 2.646 × 10^−5^). Procrustes analysis on mean shapes showed that the differences between *Ph. breyi* and *Ph. sinxayarami* rely mostly on deviations of LM Nos. 1 and 12 (Fig. S2). Plots of PCA individuals were able to discriminate between the two species, although both plots represented a small proportion of the total variance (56.7% and 47.3% for males and females, respectively) (Fig. 3).

### 3.3 MALDI-TOF

**Figure 4:**
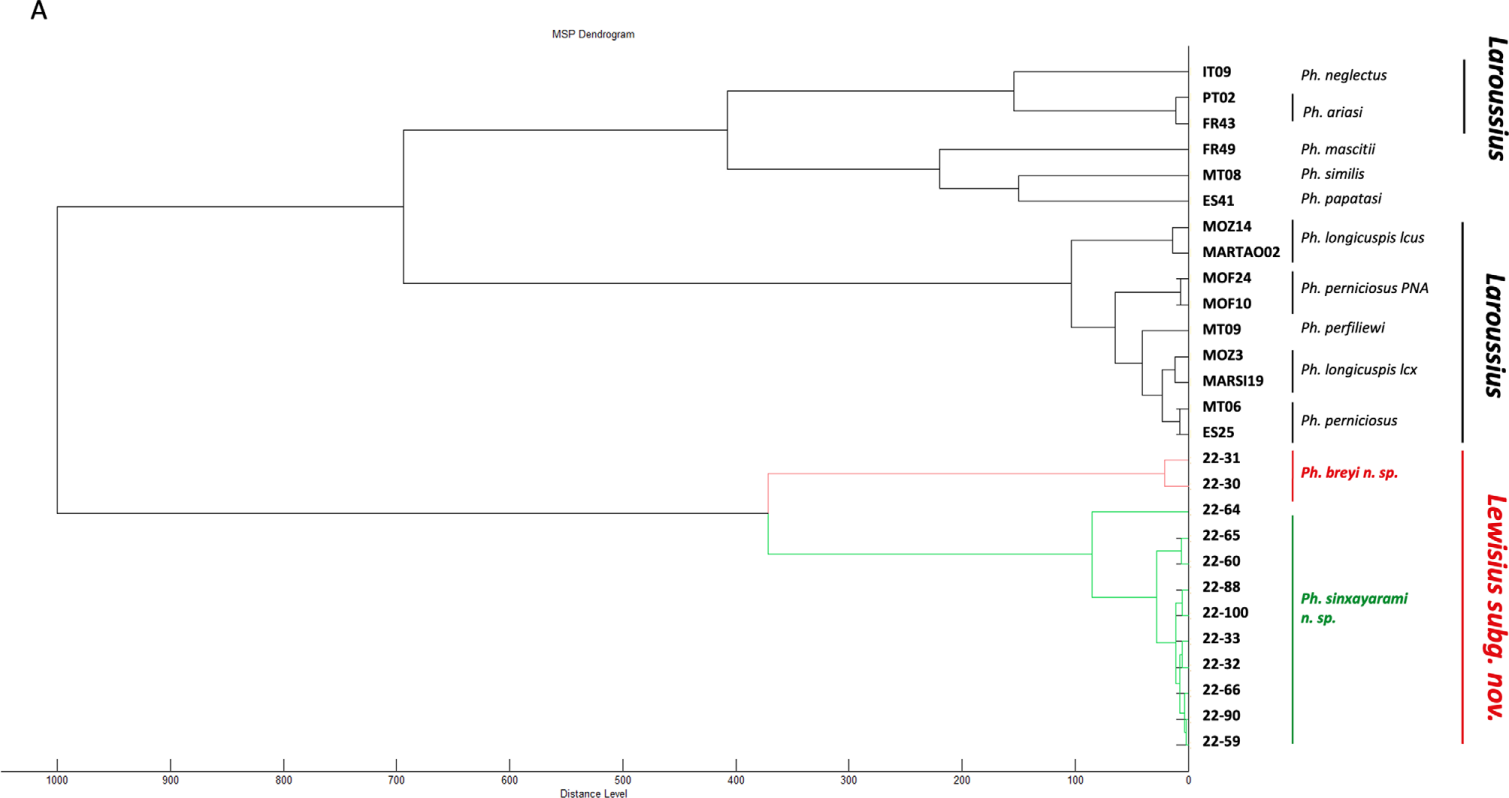
MALDI-TOF analysis. Main Spectra Profile Dendrogram using correlation distance measures and Ward algorithm for *Lewisius* n. subg. and reference spectra of our in-house database [22] of the *Larroussius* subgenus.

**Figure 5:**
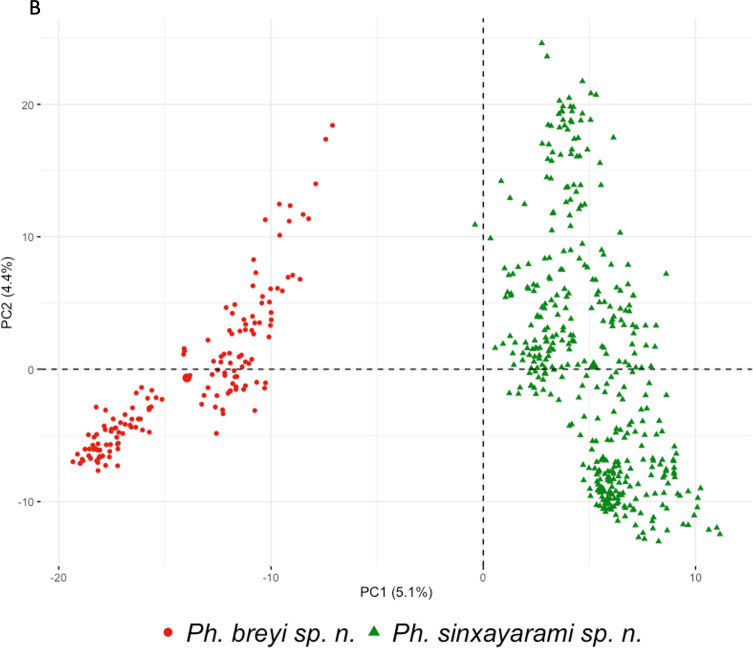
MALDI-TOF analysis. Primary Component Analysis of the spectra of *Phlebotomus* (*Lewisius*) *breyi* n. sp. and *Phlebotomus* (*Lewisius*) *sinxayarami* n. sp. Biplot of the first two principal components (PC1 and PC2).

**Figure S3:**
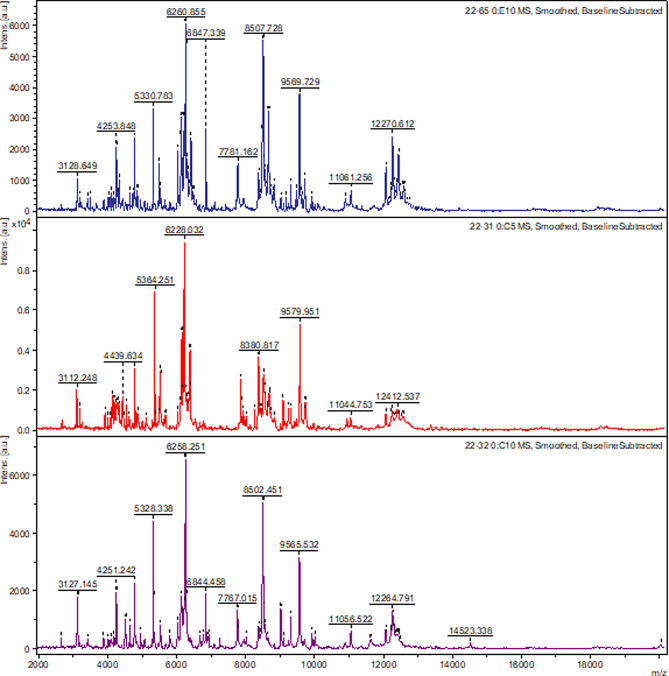
Representative MALDI-TOF spectra of *Ph. sinxayarami* n. sp. (A, C) and *Ph. breyi* n. sp. (B).

High-quality spectra were obtained from all 12 specimens of *Lewisius* n. subg. All spectra returned lower log-score values than threshold (1.7) when matched against our existing *Ph. Larroussius* group MSP database (data not shown). Hierarchical clustering (Fig. 4) allows one to (i) group all spectra (Fig. S3) from *Ph. breyi* n. sp. and *Ph. sinxayarami* n. sp. into a cluster distinct from spectra of specimens of the *Larroussius* group, and (ii) reliably discriminate between spectra from the two new species. The PCA analysis also enabled us to separate spectra from *Ph. breyi* from those of *Ph. sinxayarami* using only PCA 1 (Fig. 5), despite the low proportion of the variance explained by this component (5.1%).

### 3.4 Description of new taxa

Consensual terminology has been used in this description [18].

In our opinion, the species described below cannot be included in the subgenus *Larroussius* (see Discussion).

#### 3.4.1 Description of *Lewisius* Depaquit & Vongphayloth n. subg.

urn:lsid:zoobank.org:act:959CB10D-E575-4313-AC52-9D192608CDFB

*Genus*: *Phlebotomus* Rondani & Berté.

*Type species*: *Phlebotomus betisi* Lewis & Wharton, 1963.

*Lewisius* n. subg. is defined by (i) pharynx with armature of numerous small point-like teeth similar to those of *Larroussius*, (ii) gonostyle exhibiting five long spines out of which two are terminal, the upper external implanted subapically, the lower external implanted in the apical third and the inner one in its middle, (iii) simple parameres, (iv) conical parameral sheath, regularly tapering, and (v) segmented spermathecae, bead-like rings with a long terminal process.

*Etymology*: *Lewisius* refers to David J Lewis, Medical Entomologist at the Natural History Museum of London, who was a pioneer in the study of Phlebotomine sandflies of South-Eastern Asia and also described *Phlebotomus betisi*, the type-species of this new subgenus.

#### 3.4.2 Description of *Phlebotomus breyi* Vongphayloth & Depaquit n. sp. (Figs. 6 and 7)

urn:lsid:zoobank.org:act:4BC1E78D-8CA0-416A-9214-99A4FBA6BC39

**Figure 6:**
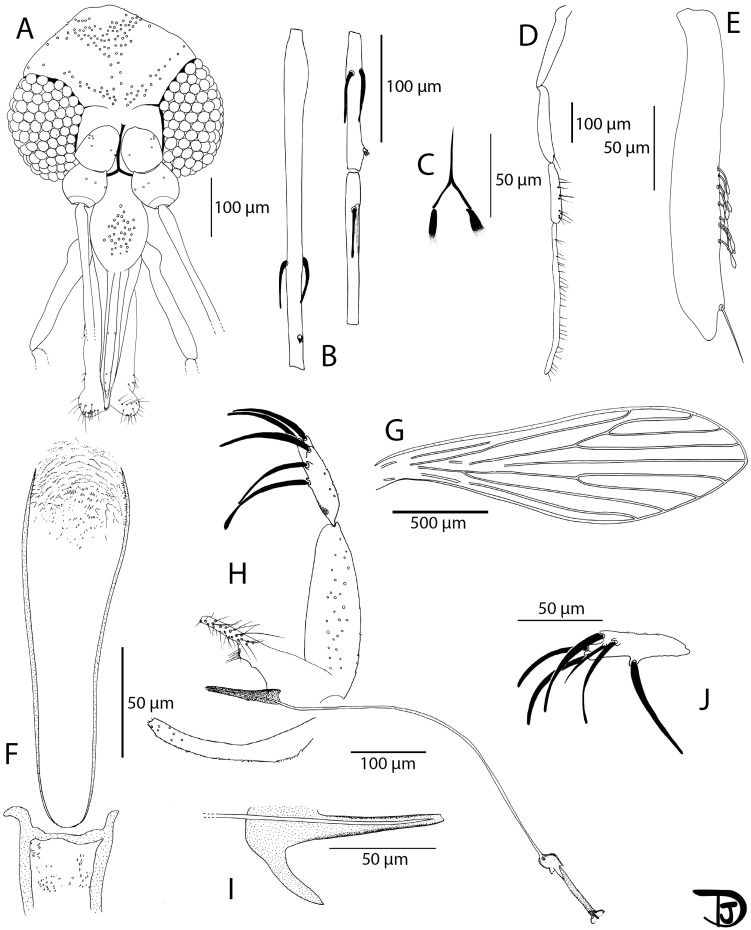
*Phlebotomus* (*Lewisius*) *breyi* n. sp. Holotype male. A: head; B: flagellomeres 1, 2 and 3 (=AIII, AIV and AV); C: labial furca; D: palp; E: third segment of the palp (P3); F: pharynx and cibarium; G: wing; H: genitalia; I: detail of the parameral sheath and the distal part of the genital ducts; and J: gonostyle exhibiting an additional thin basal sixth spine.

**Figure 7:**
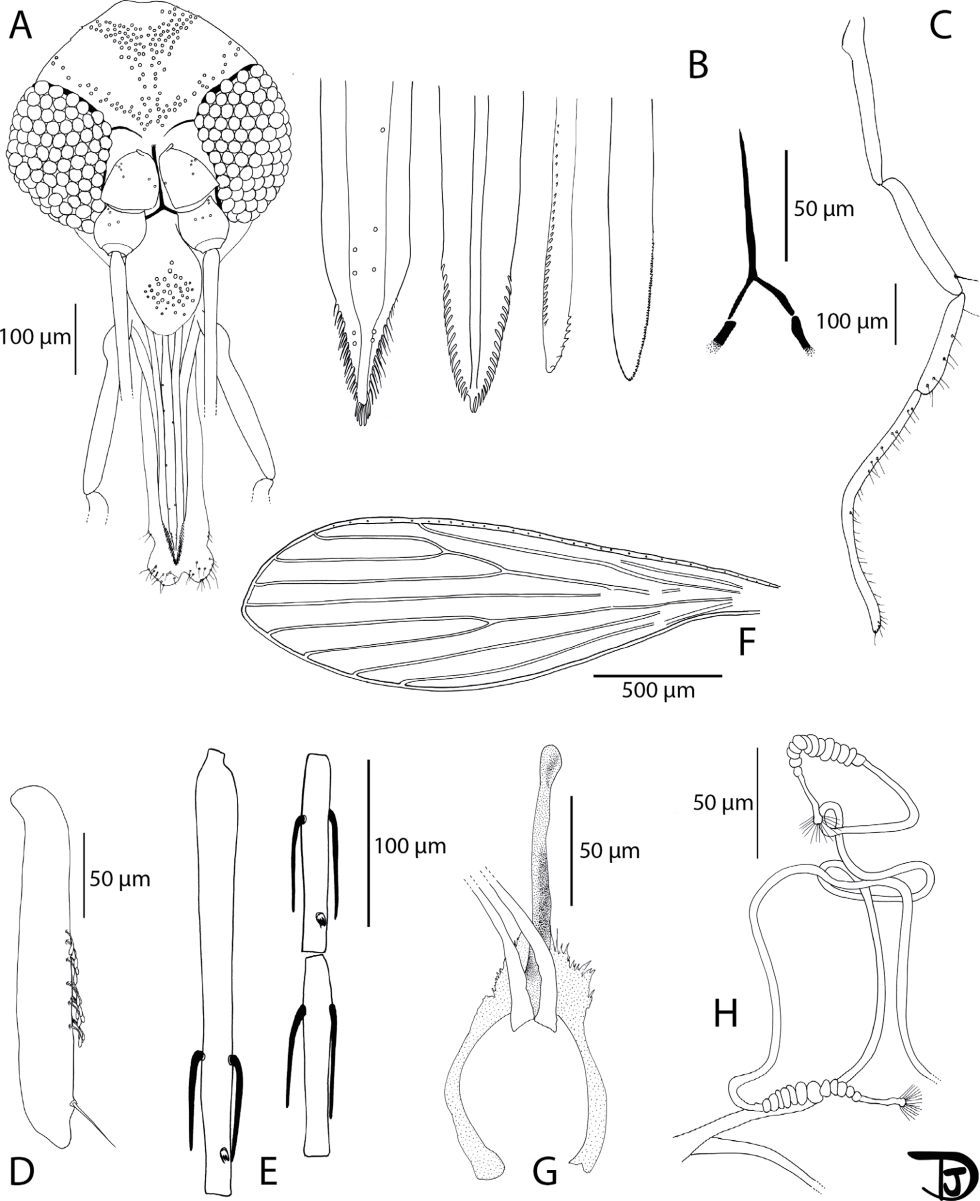
*Phlebotomus* (*Lewisius*) *breyi* n. sp. Paratype female. A: head; B: mouth parts (from left to right: labrum, hypopharynx, mandible, maxilla, and labial furca); C: palp; D: third segment of the palp; E: flagellomeres 1, 2 and 3 (=AIII, AIV and AV); F: wing; G: furca and bases of spermathecal ducts; and H: spermathecae.

*Genus*: *Phlebotomus* Rondani & Berté, in Rondani 1840.

*Subgenus*: *Lewisius* Depaquit & Vongphayloth n. subg.

*Type locality*: Pha Nok Kok cave (18°30′ N, 101°59′ E), Feung district, Vientiane province, Laos.

*Type specimens*: Holotype male (voucher LAOS 384-16) deposited in the Laboratory of Entomology of the Muséum National d’Histoire Naturelle de Paris (identification number MNHN-ED-ED11196). Two female and two male paratypes deposited at the Laboratory of Entomology of the Muséum National d’Histoire Naturelle de Paris (identification numbers MNHN-ED-ED11197, MNHN-ED-ED11198, MNHN-ED-ED11199, MNHN-ED-ED11200).

One male and one female paratype deposited at the Natural History Museum, London, UK (identification numbers NHMUK014908972 and NHMUK014908973).

One female and one male paratype deposited at the Laboratory of Medical Entomology, IPL.

*Etymology*: Epithet *breyi* refers to our Entomologist colleague Paul Brey, who created the Institut Pasteur du Laos (IPL) and the laboratory of Medical Entomology/Vector-borne diseases within the IPL.

*Note*: The authors of the new taxa are different from the authors of this paper: Article 50.1 and Recommendation 50A of the International Code of Zoological Nomenclature [24].

#### 3.4.3 Description of the male *Ph. breyi* n. sp. holotype (specimen LAO 384-16) (Fig. 6)

##### 3.4.3.1 Head (Fig. 6A)

Occiput with several lines forming a thick stripe in the posterior occiput and a narrow line along the superior part of the eyes.

Clypeus 155 μm long exhibiting 35 setae. Anterior limit difficult to observe.

Eyes: 195 μm long, 113 μm wide, with about 80 facets.

Incomplete interocular sutures.

Flagellomeres f1 (=AIII) = 289 μm, f2 (=AIV) = 133 μm, f3 (=AV) = 129 μm.

Flagellomere 1 longer than f2 + f3.

Presence of two short ascoids never reaching the next articulation from f1 to f8, 1 from f9 and 1 atrophied, 1 from F10 and f11, and no ascoid on f12–f14.

Ascoidal formula: 2/f1–f8, 1 + 1 atrophied/f9, 1/ f10–f11, 0/f12–f14.

Ascoid/f2 length ratio: 0.30

One distal papilla on f1 and f2 (Fig. 6B). Lack of papilla from f3 to f11. One papilla on f11, Four papillae on f12, four on f13, and four on f14.

No simple seta from f1 to f7. Presence of one or several simple setae from f8 to f14.

Palps (Fig. 6D): p1 = 45 μm, p2 = 167 μm, p3 = 183 μm, p4 = 150 μm, p5 = 404 μm.

Palpal formula: 1, 4, 2, 3, 5

Presence of a dozen of club-like Newstead’s sensilla on p3. No Newstead’s sensillum on other palpal segments (Fig. 6E).

Presence of one simple seta on distal p3; six on p4; more than 25 on p5. No simple seta on p1 and p2.

Labrum 244 μm long. Limit between the labrum and the clypeus difficult to observe.

Labial furca closed (Fig. 6C).

Cibarium armed with many tiny teeth pointed backwards (Fig. 6F).

Little pharyngeal teeth, commonly dot-like, sometimes pointed, and oriented backwards. All teeth are arranged along parallel curved lines.

Absence of sclerotized area.

##### 3.4.3.2 Cervix

Two cervical sensilla.

Two ventro-cervical sensilla.

##### 3.4.3.3 Thorax

560 μm long.

Light brown sclerites.

Mesonotum: post-alar setae non-observed.

Pleurae: five proepimeral setae; absence of the upper and lower anepisternal, anepimeral, metaepisternal and metaepimeral setae; presence of fine setae on the anterior region of the katepisternum, and absence of the suture between metaepisternum and katepimeron. Metafurca mounted in lateral view on all specimens.

Wings (Fig. 6G): length = 1896 μm; width = 595 μm. *r*5 = 1265 μm, *α* (*r*2) = 430 μm, *β* (*r*2 + 3) = 176 μm, *δ* = 108 μm, *γ* (*r*2 + 3 + 4) = 409 μm, *ε* (*r*3) = 581 μm, *θ* (*r*4) = 829 μm, *π* = 0 μm. Width/*γ* = 1.45.

Legs: Anterior leg: coxa = 309 μm; femur = 757 μm, tibia = 970 μm, and tarsomeres ti = 647 μm, tii–tv = 719 μm.

Median leg: coxa = 343 μm; femur = 742 μm, tibia = 1117 μm, and tarsomeres not observed.

Posterior leg: coxa = 330 μm; femur = 786 μm, tibia = 1378 μm, tarsomeres ti = 798 μm, tii–tv = 827 μm.

##### 3.4.3.4 Abdomen

Tergites ii–v: presence of randomly distributed setae.

Tergites ii–vii: absence of tergal papillae.

##### 3.4.3.5 Genitalia (Fig. 6H)

Absence of abdominal rods.

Gonocoxite: 223 μm long, 68 μm width, with randomly distributed internal setae, without any tuft. Absence of basal gonocoxal lobe.

Gonostyle: 117 μm long with 5 thick spines (two terminal ones, the superior external implanted subapically, the inferior external situated in the apical third and the internal in its middle). Presence on the holotype, as well as on another specimen, of a gonostyle exhibiting an additional thin basal sixth spine (Fig. 6J).

Absence of accessory setae.

Simple paramere 189 μm long with a slight tubercle carrying about 6 setae on its lower side.

Absence of accessory spine between the paramere and the parameral sheath.

Parameral sheath: 100 μm straight, with a blunt end at its top (Fig. 6I).

Aedeagal ducts: 561 μm long, isodiametric, pointed at their top. Sperm pump 108 μm long. Ejaculatory apodeme 96 μm long.

Epandrial lobes: 225 μm long, about as long as the gonocoxites, without permanent setae.

#### 3.5.1 Description of the female *Ph. breyi* n. sp. paratype (specimen LAOS#251 type1) (Fig. 7)

##### 3.5.1.1 Head (Fig. 7A)

Occiput with several lines forming a thick stripe in the posterior occiput and a narrow line along the superior part of the eyes.

Clypeus 178 μm long, exhibiting 47 setae. Anterior limit difficult to observe.

Eyes: 223 μm long, 128 μm wide.

Incomplete interocular sutures.

Flagellomeres (Fig. 7E) f1 (=AIII) = 293 μm, f2 (=AIV) = 129 μm, f3 (=AV) = 126 μm.

Flagellomere 1 longer than f2 + f3.

Presence of 2 short ascoids never reaching the next articulation from f1 to f13.

Ascoidal formula: 2/f1 − f13.

Ascoid/f2 length ratio: 0.57

One distal papilla on f1 and f2 (Fig. 7E). Lack of papilla from f3 to f11. One papilla on f1. One on f2. No papilla on f3 to f10. One papilla on f11. Five on f12 and f13. And four on f14.

No simple seta from f1 to f7. Presence of one or several simple setae from f9 to f14.

Palps (Fig. 7C): p1 = 51 μm, p2 = 213 μm, p3 = 218 μm, p4 = 173 μm, p5 = 413 μm.

Palpal formula: 1, 4, (2, 3), 5

Presence of about 15 club-like Newstead’s sensilla on p3 (Fig. 7D). No Newstead’s sensillum on other palpal segments.

Presence of one simple seta on distal p3; seven on p4; about 40 on p5. No simple seta on p1 and p2.

Labrum 309 μm long. Limit between the labrum and the clypeus difficult to observe.

Labial furca closed (Fig. 7B).

Cibarium armed with many tiny teeth pointed backwards.

Little pharyngeal teeth, commonly dot-like, sometimes pointed and oriented backwards. All teeth are arranged along parallel curved lines.

Absence of sclerotized area.

##### 3.5.1.2 Cervix

Two cervical sensilla.

Ventro-cervical sensilla not observed.

##### 3.5.1.3 Thorax

654 μm long.

Light brown sclerites.

Mesonotum: post-alar setae non-observed.

Pleurae: five proepimeral setae; absence of the upper and lower anepisternal, anepimeral, metaepisternal and metaepimeral setae; presence of fine setae on the anterior region of the katepisternum, and absence of the suture between metaepisternum and katepimeron. Metafurca mounted in lateral view on all specimens.

Wings (Fig. 7F): length = 1690 μm; width = 564 μm. *r*5 = 1139 μm, *α* (*r*2) = 398 μm, *β* (*r*2 + 3) = 174 μm, *δ* = 82 μm, *γ* (*r*2 + 3+4) = 332 μm, *ε* (*r*3) = 532 μm, *θ* (*r*4) = 789 μm. Width/*γ* = 1.70.

##### 3.5.1.4 Abdomen

Tergites ii–v: presence of randomly distributed setae.

Tergite VIII and IX not observed.

Cerci 133 μm long.

Setae were not observed on the X sternite.

##### 3.5.1.5 Genitalia (Figs. 7G and 7H)

Spermathecae were measured and drawn in Marc-André solution on specimen 356-1 before its remounting in CMCP9 medium. Smooth and thin wall individual ducts 450 μm long (Fig. 7H). Ducts are isodiametric except for a basal slight enlargement (Fig. 7G). Annealed spermathecae with 12 or 13 bead-like rings. Terminal knob (head) carried by a long and narrow neck.

Genital fork without lateral apodemes.

#### 3.5.2 Description of *Phlebotomus sinxayarami* Vongphayloth & Depaquit n. sp. (Figs. 8 and 9)

urn:lsid:zoobank.org:act:21E34424-6F37-4522-8844-BBAECA6A798A

**Figure 8:**
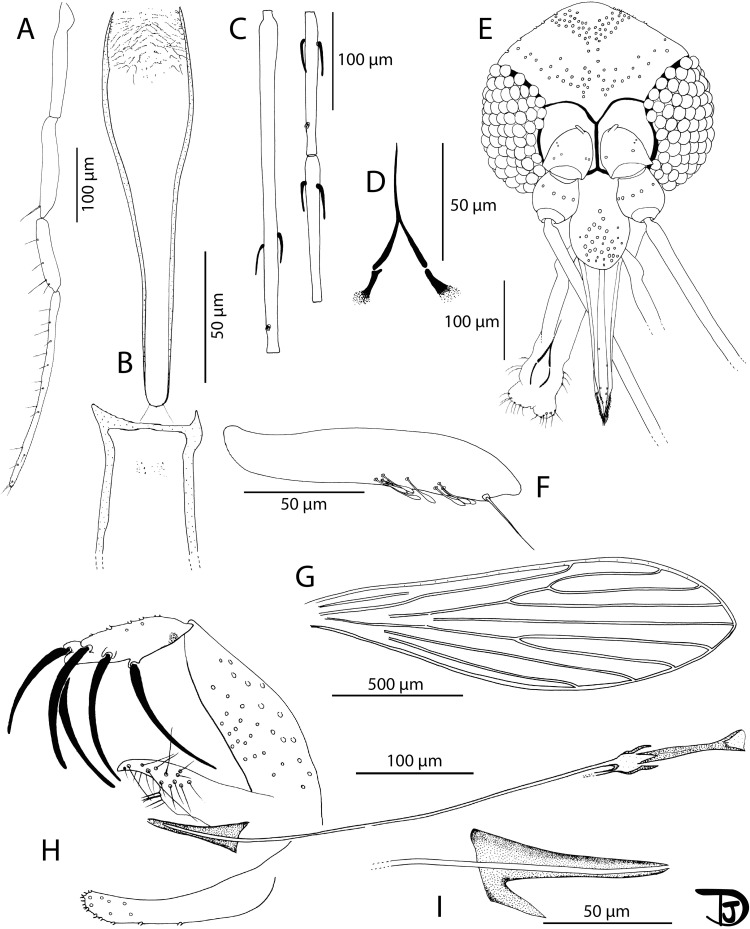
*Phlebotomus* (*Lewisius*) *sinxayarami* n. sp. Holotype male. A: palp; B: pharynx and cibarium; C: flagellomeres 1, 2 and 3 (=AIII, AIV and AV); D: labial furca; E: head; F: third segment of the palp (P3); G: wing; and H: genitalia.

**Figure 9:**
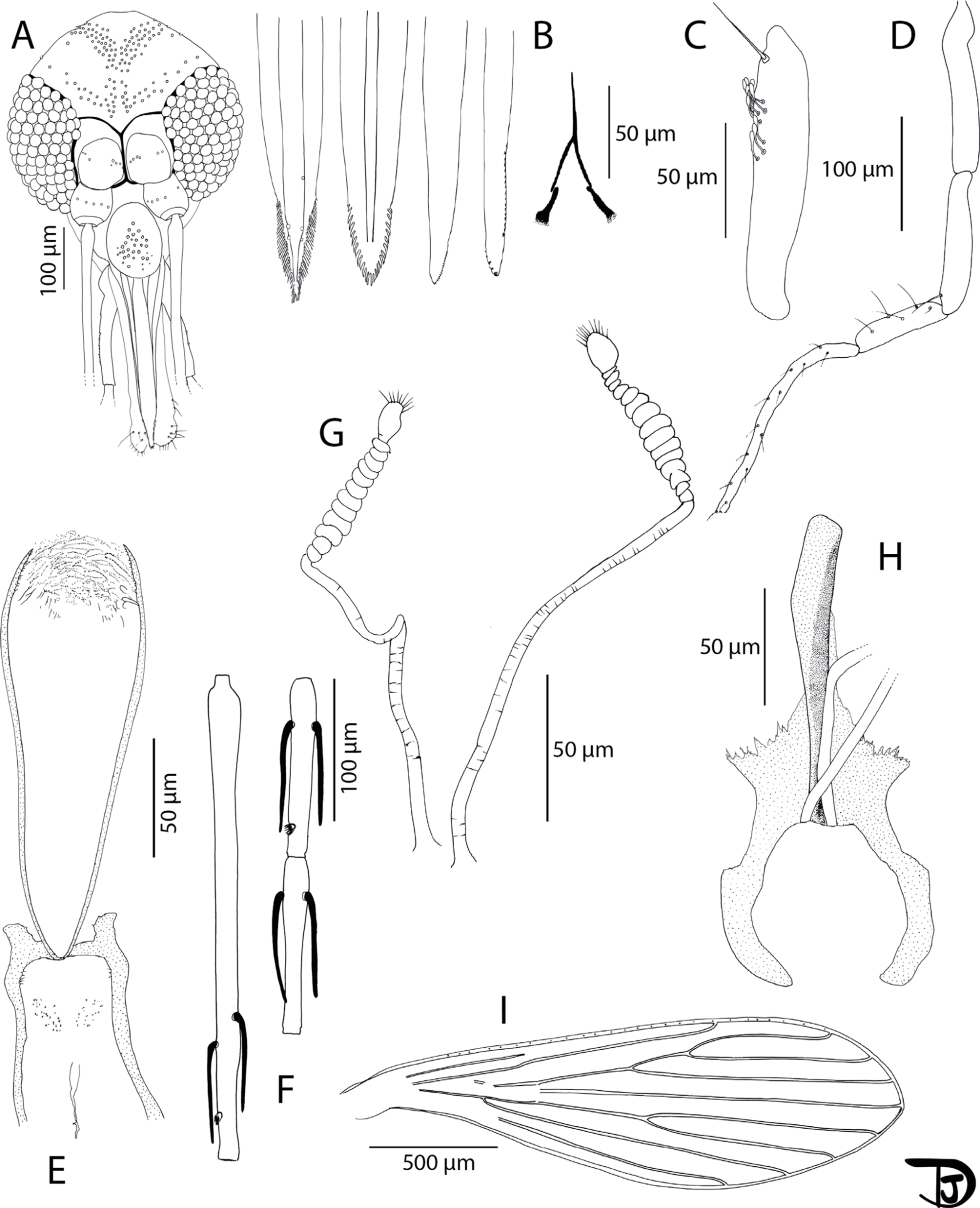
*Phlebotomus* (*Lewisius*) *sinxayarami* n. sp. Paratype female. A: head; B: mouth parts (labrum, hypopharynx, mandible, maxilla, and labial furca from left to right); C: third segment of the palp (P3); D: palp; E: pharynx and cibarium; F: flagellomeres 1, 2 and 3 (=AIII, AIV and AV); G: spermathecae; H: furca and bases of spermathecal ducts; and I: wing.

Genus *Phlebotomus* Rondani & Berté, in Rondani 1840.

Subgenus *Lewisius* Depaquit & Vongphayloth n. subg.

*Type locality*: Pha Nok Kok cave (18°30′ N, 101°59′ E), Feung district, Vientiane province, Laos.

*Type specimens*: Holotype male (voucher NGS 356-17) deposited at the Laboratory of Entomology of the Muséum National d’Histoire Naturelle de Paris (identification number MNHN-ED-ED11201). Two female and two male paratypes deposited at the Laboratory of Entomology of the Muséum National d’Histoire Naturelle de Paris (identification numbers MNHN-ED-ED11202, MNHN-ED-ED11209, MNHN-ED-ED11210, MNHN-ED-ED11211).

One male and one female paratype deposited at the Natural History Museum of London, UK (identification numbers NHMUK014908974 and NHMUK014908975). One female and one male paratype deposited at the Laboratory of Entomology of Institut Pasteur du Laos.

*Etymology*: Epithet *sinxayarami* refers to the Sinxayaram temple located close to the cave where the type specimens have been caught.

*Note*: The authors of the new taxa are different from the authors of this paper: Article 50.1 and Recommendation 50A of the International Code of Zoological Nomenclature [24].

#### 3.5.3 Description of the male *Ph. sinxayarami* n. sp. holotype (specimen NGS 356-17)

#### 3.5.3.1 Head (Fig. 8E)

Occiput with several lines forming a thick stripe in the posterior occiput and a narrow line along the superior part of the eyes.

Clypeus 122 μm long, exhibiting 35 setae. Anterior limit difficult to observe.

Eyes 183 μm long, 95 μm wide.

Complete interocular sutures.

Flagellomeres (Fig. 8C) f1 (=AIII) = 342 μm, f2 (=AIV) = 148 μm, f3 (=AV) = 147 μm.

Flagellomere 1 longer than f2 + f3.

Presence of two very short ascoids never reaching the next articulation from f1 to f9, 1 from f10 to f11, and no ascoid on f12 to f14.

Ascoidal formula: 2/f1–f9, 1/f10–f11, 0/f12–f14.

Length ratio: Ascoid/f2 = 0.23

One distal papilla on f1 and f2 (Fig. 8C). Lack of papilla from f3 to f11. One papilla on f1 and f2. Three on f11. Four papillae on f12, five papillae on f12 to f14.

No simple setae from f1 to f7. Presence of one or several simple setae from f8 to f14.

Palps (Fig. 8A): p1 = 37 μm, p2 = 108 μm, p3 = 122 μm, p4 = 86 μm, p5 = 270 μm.

Palpal formula: 1, 4, 2, 3, 5

Presence of 8 club-like Newstead’s sensilla on p3 (Fig. 8F). No Newstead’s sensillum on other palpal segments.

Presence of one simple seta on distal p3; four simple setae on p4; about 15 on p5. No simple seta on p1 and p2.

Labrum 191 μm long. Limit between the labrum and the clypeus difficult to observe.

Labial furca closed (Fig. 8D).

Cibarium armed with many tiny dot-like teeth, difficult to observe, despite the use of phase contrast (Fig. 8B).

Little pharyngeal teeth, commonly dot-like, sometimes pointed, and oriented backwards. All teeth are arranged along parallel curved lines.

Absence of sclerotized area.

##### 3.5.3.2 Cervix

Two cervical sensilla.

Ventro-cervical sensilla not observed.

##### 3.5.3.3 Thorax

513 μm long.

Light brown sclerites.

Mesonotum: post-alar setae non-observed.

Pleurae: five proepimeral setae; absence of the upper and lower anepisternal, anepimeral, metaepisternal and metaepimeral setae; presence of fine setae on the anterior region of the katepisternum, and absence of the suture between metaepisternum and katepimeron. Metafurca mounted in lateral view on all specimens.

Wings (Fig. 8G): length = 1619 μm; width = 475 μm. *r*5 = 1156 μm, *α* (*r*2) = 434 μm, *β* (*r*2 + 3) = 149 μm, *δ* = 108 μm, *γ* (*r*2 + 3 + 4) = 298 μm, *ε* (*r*3) = 572 μm, *θ* (*r*4) = 814 μm. Width/*γ* = 1.60.

##### 3.5.3.4 Abdomen

Tergites ii–v: presence of randomly distributed setae.

Tergites ii–vii: absence of tergal papillae.

##### 3.5.3.5 Genitalia (Fig. 8H)

Absence of abdominal rods.

Gonocoxite: 204 μm long, 58 μm width, with randomly distributed internal setae, without any tuft. Absence of basal gonocoxal lobe.

Gonostyle: 105 μm long with 5 thick spines (two terminal ones, two median ones and an intermediate one).

Absence of accessory setae.

Simple paramere: 140 μm long with a slight tubercle carrying about 5 setae on its lower side.

Absence of accessory spine between the paramere and the parameral sheath.

Parameral sheath: 100 μm straight, rounded at its top.

Aedeagal ducts, 390 μm long, isodiametric, pointed at their top. Sperm pump 98 μm long. Ejaculatory apodeme 96 μm long.

Epandrial lobes: 196 μm long, about as long as the gonocoxites, without permanent setae.

#### 3.5.4 Description of the female *Ph. sinxayarami* n. sp. paratype (specimen NGS 356-29) (Fig. 9)

##### 3.5.4.1 Head (Fig. 9A)

Occiput with several lines forming a thick stripe in the posterior occiput and a narrow line along the superior part of the eyes.

Clypeus 138 μm long, exhibiting 35 setae. Anterior limit difficult to observe.

Eyes 179 μm long, 97 μm wide.

Complete interocular sutures.

Flagellomeres (Fig. 9F) f1 (=AIII) = 301 μm, f2 (=AIV) = 121 μm, f3 (=AV) = 112 μm.

Flagellomere 1 longer than f2 + f3.

Presence of 2 short ascoids never reaching the next articulation from f1 to f11, probably two but the second one is not visible from f12 and no ascoid on f13–f14.

Ascoidal formula: 2/f1–f11, 1(1)/f12, 0/f13.

Ascoid/f2 length ratio: 0.56

One distal papilla on f1 and f2. No papilla from f3 to f9. One on f10. Three on f11 to f13. Four papillae on f12, four on f13.

No simple seta from f1 to f7. Presence of one or several simple setae from f9 to f14.

Palps (Fig. 9D): p1 = 37 μm, p2 = 120 μm, p3 = 128 μm, p4 = 923 μm, p5 = 238 μm.

Palpal formula: 1, 4, (2, 3), 5

Presence of about 15 club-like Newstead’s sensilla on p3 (Fig. 9C). No Newstead’s sensilla on other palpal segments.

Presence of one simple seta on distal p3; seven simple setae on p4; about 40 on p5. No simple seta on p1 and p2.

Labrum 214 μm long. Limit between the labrum and the clypeus difficult to observe.

Labial furca closed (Fig. 9B).

Cibarium armed with many tiny teeth pointed backwards (Fig. 9E).

Little pharyngeal teeth, commonly dot-like, sometimes pointed and oriented backwards. All teeth are arranged along parallel curved lines.

Absence of sclerotized area.

##### 3.5.4.2 Cervix

Two cervical sensilla.

Ventro-cervical sensilla not observed.

##### 3.5.4.3 Thorax

521 μm long.

Light brown sclerites.

Mesonotum: post-alar setae non-observed.

Five proepimeral setae.

Pleurae: five proepimeral setae; absence of the upper and lower anepisternal, anepimeral, metaepisternal and metaepimeral setae; presence of fine setae on the anterior region of the katepisternum, and absence of the suture between metaepisternum and katepimeron. Metafurca mounted in lateral view on all specimens.

Wings (Fig. 9I): length = 1752 μm; width = 588 μm. *r*5 = 1266 μm, *α* (*r*2) = 535 μm, *β* (*r*2 + 3) = 167 μm, *δ* = 163 μm, *γ* (*r*2 + 3+4) = 304 μm, *ε* (*r*3) = 674 μm, *θ* (*r*4) = 926 μm, *π* = 0 μm. Width/*γ* = 1.93.

##### 3.5.4.4 Abdomen

Tergites ii–v: presence of randomly distributed setae.

About 20 setae on tergite VIII.

Absence of protuberance on tergite IX.

Cerci 118 μm long.

Setae not observed on the X sternite.

##### 3.5.4.5 Genitalia (Figs. 9G and 9H)

Spermathecae were measured and drawn in Marc-André solution on specimen 356-1 before its remounting. Smooth and thin wall individual ducts 160 μm long. Ducts are isodiametric and enlarged at their bases (Fig. 9H). Annealed spermathecae with 14 or 15 bead-like rings. Terminal knob (=head) inserted in a wide neck (Fig. 9G).

Genital fork with short lateral apodemes.

Tables 4 and 5 summarize the measurements carried out on several male and female specimens of *Ph. breyi* n. sp., *Ph. sinxayarami* n. sp., and *Ph. betisi*.

**Table 4:** Measurement of females of three species: *Ph. breyi* n. sp., *Ph. sinxayarami* n. sp., and *Ph. betisi* from Malaysia.

	*Ph. breyi* n. sp.					*Ph. sinxayarami* n. sp.					*Ph. betisi*				
	No. of specimens	Max	Min	Average	Standard deviation	No. of specimens	Max	Min	Average	Standard deviation	No. of specimens	Max	Min	Average	Standard deviation
Wing															
Length	9	2164.33	1695.71	2000.31	160.08	10	2239.45	1707.52	1970.33	147.90	2	2098.49	1928.28	2013.39	120.36
Width	9	696.96	565.15	640.79	46.95	10	658.73	490.26	598.20	48.96	3	670.45	624.79	640.07	26.31
Alpha (R2) α	9	557.30	387.67	469.89	57.09	10	591.52	397.13	514.80	54.60	3	600.14	510.61	552.44	45.05
R3	9	730.64	533.28	640.46	65.00	10	930.24	547.78	682.38	99.96	3	757.67	658.38	716.25	51.65
R4	9	1048.92	799.16	931.16	80.57	10	1050.49	804.78	947.41	67.15	3	1007.40	897.49	960.02	56.50
R5	9	1489.49	1124.37	1342.68	117.66	10	1482.92	1141.07	1304.20	91.42	3	1417.85	1306.74	1378.02	61.87
Beta (R2 + R3) β	9	226.07	169.76	201.51	17.55	10	243.85	158.83	203.36	30.46	3	183.02	153.49	171.82	16.01
Delta (R2 + 3-R1) δ	9	167.29	72.67	112.23	33.21	10	182.15	52.49	110.00	34.96	3	215.31	168.39	194.75	23.99
Gamma (R2 + 3+4)	9	440.33	321.69	384.19	40.27	10	408.74	275.87	322.91	38.15	3	409.27	374.13	388.13	18.63
Pi (R2 + 3-M1 + 2)	9	59.22	9.51	29.13	15.87	10	113.23	20.18	71.62	30.97	3	66.06	32.29	52.09	17.62
R2/R2 + 3	9	2.63	1.93	2.34	0.23	10	3.26	2.02	2.58	0.44	3	3.35	2.99	3.22	0.20
Head															
f1 (AIII)	5	293.87	256.01	276.80	14.95	10	346.48	253.05	310.18	25.91	3	352.87	309.06	328.91	22.19
f2 (AIV)	5	134.90	113.78	123.19	8.33	10	136.21	105.74	122.48	8.25	3	143.06	132.07	136.65	5.72
f3 (AV)	4	125.56	111.63	120.07	6.35	10	137.78	109.65	124.17	8.08	3	135.18	128.70	132.30	3.30
f2 + f3 (AIV + AV)	4	260.46	225.41	244.22	15.48	10	273.99	215.39	246.65	16.17	3	278.24	260.77	268.96	8.79
f1/f2 + f3 (AIII/AIV + AV)	4	1.20	1.11	1.14	0.04	10	1.33	1.17	1.26	0.05	3	1.27	1.19	1.22	0.04
Clypeus	9	182.93	130.08	167.78	17.08	10	143.69	123.45	132.39	5.70	3	161.20	153.89	157.39	3.66
Labrum	9	320.26	275.54	304.78	16.33	10	284.85	205.14	249.16	23.52	3	311.61	293.77	300.25	9.87
F1 (AIII)/labrum	5	0.92	0.87	0.89	0.02	10	1.38	1.12	1.25	0.08	3	1.19	1.04	1.10	0.09
P1	10	60.21	39.45	50.31	6.75	10	39.07	33.04	36.37	2.16	3	55.68	45.35	49.27	5.59
P2	10	218.32	165.30	194.29	17.17	10	155.27	112.01	135.61	11.18	3	215.06	193.45	205.74	11.11
P3	10	216.12	173.41	195.10	13.32	10	147.11	121.12	135.82	9.88	3	209.92	178.74	198.40	17.11
P4	10	172.48	141.01	154.12	12.74	10	105.16	80.58	92.03	8.11	3	140.66	126.39	135.84	8.18
P5	9	426.75	308.15	372.52	47.93	10	293.55	135.30	212.32	44.45	3	452.85	393.31	428.49	31.21

**Table 5:** Measurement of males of three species: *Ph. breyi* n. sp., *Ph. sinxayarami* n. sp., and *Ph. betisi* from Malaysia

	*Ph. bre yi* n. sp.					*Ph. sinxayarami* n. sp.					*Ph. betisi*				
	No. of specimens	Max	Min	Average	Standard deviation	No. of specimens	Max	Min	Average	Standard deviation	No. of specimens	Maxi	Min	Average	Standard deviation
Wing															
Length	9	1968.57	1740.77	1877.49	77.67	7	1721.45	1547.89	1649.50	60.22	8	1744	1596	1683	55.7
Width	10	631.18	546.19	588.13	26.28	7	544.03	461.90	500.79	25.11	9	522	483	504	13.3
Alpha (R2) a	10	472.32	391.64	439.02	24.10	7	445.45	388.89	419.55	21.82	9	472	401	435	21.3
R3	10	645.88	560.07	591.24	28.71	7	589.38	512.09	557.18	25.50	–	–	–	–	–
R4	10	916.34	827.67	855.10	29.01	7	864.86	757.65	805.96	37.81	–	–	–	–	–
R5	10	1321.36	1225.78	1259.27	36.48	7	1172.98	1045.51	1116.89	45.16	–	–	–	–	–
Beta (R2 + R3) b	10	200.64	170.93	183.35	8.95	7	178.96	146.72	165.82	13.39	9	155	113	135	14.4
Delta d (R2 + 3 − R1)	10	151.00	64.64	102.59	24.25	7	91.54	45.77	73.27	17.75	9	298	96	164	63.6
Gamma (R2 + 3 + 4) g	10	410.62	331.05	370.65	21.36	7	304.14	227.50	274.38	25.60	9	455	279	334	52.6
Pi p (R2 + 3-M1 + 2)	10	63.34	6.73	29.63	14.81	7	86.21	33.64	57.36	20.17	9	103	14	54	30.9
R2/R2 + 3	10	2.57	2.09	2.40	0.14	7	2.90	2.35	2.54	0.19	–	–	–	–	–
Head															
f1 (AIII)	8	330.98	268.11	303.08	21.33	7	334.08	239.33	301.88	34.88	10	347	298	328	15.6
f2 (AIV)	7	146.28	130.56	137.93	6.65	7	149.48	122.03	135.36	9.41	10	153	142	148	4.5
f3 (AV)	7	141.93	128.48	135.08	5.15	7	144.94	119.95	134.97	8.05	10	149	135	142	4.8
f2 + f3 (AIV + AV)	7	286.02	259.70	273.01	10.54	7	294.42	241.98	270.33	17.27	10	302	278	290	9.01
Clypeus	10	164.73	142.70	154.60	6.71	7	125.67	109.15	119.94	5.26	–	–	–	–	–
Labrum	10	261.69	238.06	250.50	8.65	7	213.48	192.18	202.89	7.60	10	267	208	242	18.9
f1/f2 + f3 (AIII/AIV + AV)	7	1.18	1.03	1.11	0.05	7	1.23	0.88	1.12	0.12	10	1.17	1.07	1.13	0.03
f1 (AIII)/labrum	10	1.31	0.00	0.97	0.52	7	1.69	1.21	1.49	0.17	10	1.52	1.18	1.36	0.1
P1	9	53.09	36.97	43.47	4.86	8	35.31	30.84	32.52	1.51	10	60	34	45	7.6
P2	9	186.87	136.69	174.76	15.10	8	129.24	101.22	115.96	9.53	10	215	150	195	21.7
P3	8	188.41	174.74	180.33	4.86	8	130.99	103.68	119.59	9.41	9	232	165	182.3	19.9
P4	8	153.53	136.26	142.99	6.41	8	93.46	66.40	84.05	8.07	9	121	99	108.1	7.5
P5	6	424.41	292.33	364.61	54.43	8	272.04	182.64	233.49	32.05	9	380	308	348	27.4
Genitalia															
Parameral sheath	10	106.28	96.92	101.13	2.32	8	85.59	74.00	79.99	4.19	9	133	93	111	11.7
Gonostyle	10	117.08	103.20	111.74	4.00	8	111.54	99.20	103.98	4.04	9	117	102	109	4.3
Gonocoxite	10	229.16	207.78	220.34	7.39	8	204.76	183.86	196.20	8.06	9	224	168	193	15.7
Sperm pump (SP)	10	116.51	95.83	107.49	6.32	8	110.63	92.17	100.76	5.55	9	129	112	121	4.8
Paramere	10	196.99	160.00	182.62	12.92	8	153.32	138.06	142.47	4.92	9	187	153	170	10.3
Surstyle	10	237.98	198.33	216.01	13.85	8	195.37	172.85	187.13	7.84	9	218	179	198	12
Aedeagal ducts (AD)	10	655.39	535.60	599.87	34.11	8	411.75	328.91	374.47	26.02	9	470	331	362	39.7
Gonocoxite/gonostyle	10	2.15	1.88	1.97	0.10	8	2.00	1.81	1.89	0.07	10	2.02	1.44	1.78	0.17
AD/SP	10	6.25	5.03	5.59	0.39	8	4.02	3.35	3.72	0.26	10	3.92	2.74	3.01	0.35

## 4 Discussion

The new species status given to *Ph. breyi* n. sp. and *Ph. sinxayarami* n. sp. is very strong. It is based on morphologic, morphometric, geomorphometric, molecular and proteomic arguments even though this last approach was not possible on the specimens of *Ph. betisi* in our possession that were all preserved in alcohol before being mounted. The association of males and females, in addition to the fact that each of the specimens are sympatric, having all been captured in the same cave, is based on the same evidence, regardless of the approach considered.

From a morphological point of view, the three species are easily individualized.

In both sexes, *Ph. betisi* and *Ph. breyi* do not have a complete interocular suture while *Ph. sinxayarami* owns one. This character is very unusual in the genus *Phlebotomus* [38] and is rather observed in the American genera *Warileya* [18]. In the Old World, the genus *Chinius* also shares a complete interocular suture [15].

For males, the main discriminating character (Tables 4, 5 and 6) between these three species is the f1/f2 + 3 ratio. This ratio is low in *Ph. breyi* n. sp., intermediate in *Ph. sinxayarami* and high for *Ph. betisi*. There is no overlap between the three species for this trait. Moreover, the length of the aedeagal ducts is discriminating: they are very long in *Ph. breyi* (>478 μm), long in *Ph. sinxayarami* (331–470 μm) and short in *Ph. betisi* (311–387 μm). Similarly, the palps (particularly p4 and p5) are short in *Ph. sinxayarami* while they are long in *Ph. breyi* and *Ph. betisi*.

**Table 6:** Differential diagnosis of the three presently known species of *Lewisius* n. subg.

Gender and character			
	*Ph. betisi* Lewis & Wharton	*Ph. sinxayarami* n. sp.	*Ph. breyi* n. sp.
Females			
ascoid formula antennal papillae f1 (AIII)/labrum palp segment 4 spermathecae length of spermathecal individual ducts	2/f1–f13, 0/f14 Presence on f1–f2, f9–f14, and absence on f3–f8 > 1 < 141 (126–141) about 20 clearly separated rings with long neck < 400, No common duct	2/f1–f13, 0/f14 Presence on f1–f2, f10–f14, and absence on f3–f9 > 1 < 141 (80–110) about 12–13 clearly separated rings with short neck < 400, No common duct	2/f1–f13, 0/f14 Presence on f1–f2, f12–f14, and absence on f3–f11 < 1 > 141 (141–173) about 14–15 clearly separated rings with long neck > 400, No common duct
Males			
ascoid formula antennal papillae Aedeagal ducts aedeagal ducts/sperm pump	2/f1–f6, 1/f7–f12, 0/f13–f14 Presence on f1–f2, f9–f14, and absence on f3–f8 < 500 < 4	2/f1–f9, 1/f10–f11, 0/f12–f14 Presence on f1–f2, f10–f14, and absence on f3–f9 < 500 μm < 4	2/f1–f9, 1/f10–f11, 0/f12–f14 Presence on f1–f2, f12–f14, and absence on f3–f11 > 500 μm > 4

For females, the length of the palps, particularly p4 and p5 are short in *Ph. Sinxayarami*, while they are long in *Ph. breyi* and *Ph. betisi*. The spermathecal ducts of *Ph. sinxayarami* are longer than those of *Ph. breyi.* The distal part of the spermatheca bearing the head is similar to a neck and thin in *Ph. breyi* and in *Ph. betisi*, while it is enlarged in *Ph. sinxayarami.*

Until now, *Ph. betisi* was the only *Phlebotomus* from Southeast Asia to exhibit in the male a gonostyle bearing five spines associated with a simple paramere, differing from the *Phlebotomus* of the subgenus *Euphlebotomus* having a five-spine gonostyle associated with a complex paramere. Females were the only ones of the genus *Phlebotomus* in this same region to have a pharyngeal framework consisting of small punctiform teeth and annealed spermathecae whose head is carried by a neck like that observed in females of the subgenus *Larroussius*. This exclusivity in the species described so far prompted the authors to quickly identify the specimens possessing these characters as being *Ph. betisi*. Paradoxically, this great originality of the characters may have hidden specific diversity which would henceforth prompt us to review all the mentions of *Ph. betisi* in the literature, starting with those having been used to describe the males, although separated only by about 20 kilometres from the type-locality in Malaysia. A checking of the specimens recorded in Thailand [44] or Vietnam [48, 49] is now necessary. Additionally, several records of *Ph. major* in Southeast Asia [5–7, 23, 32–35, 44] are probably irrelevant and certainly refer to *Ph. betisi*. We recommend their re-examination.

Interestingly, both males and females did not exhibit any papilla on the 3rd flagellomere. This is completely unusual regarding the genus *Phlebotomus*. If old descriptions did not mention the record/absence of such papilla on the 3rd flagellomere, recent observations carried out on the genera *Phlebotomus* and *Sergentomyia* highlighted every time the presence of such papilla on the 3rd flagellomere for members of the genus *Phlebotomus*, whereas the species belonging to the genus *Sergentomyia* never exhibits such papilla [11, 13, 16, 39, 40]. This unusual observation means that all species belonging to the subgenus *Lewisius* n. subg. share a character previously observed in the genus *Sergentomyia*.

The position of *Ph. betisi*, *Ph. breyi*, and *Ph. sinxayarami* in the subgenus *Larroussius* does not seem relevant to us. When describing the first species, Lewis & Wharton [29] did not classify *Ph. betisi* in the subgenus *Larroussius* explaining about its systematic position: “the spermatheca shows that this species may belong to the group of *Ph. major* Annandale (subgenus *Larroussius* of Theodor, 1948, 1958), but the bead-like segments and narrowness of the process are unusual. The discovery of the male would probably clarify the position of *Ph. betisi*”. In 1978, Lewis [28] initially classified *Ph. betisi* in the subgenus *Larroussius* then followed by several authors reviewing the classification of sandflies [8, 9, 27, 43]. When they described the male, Khadri et al. [25] indicated that within the *Larroussius* subgenus, two distal and three median spines on the style were usually present. *Phlebotomus betisi* differed from other *Larroussius* males by the position of these spines: two distal, one between these and the two intermediate ones. This character is sometimes shared by *Phlebotomus* belonging to the subgenus *Euphlebotomus*. These morphological characters could thus be sufficient to support the validity of the subgenus *Lewisius* n. subg. Moreover, the molecular approach (Fig. 2) very clearly individualizes the three species of *Lewisius* revealing the monophyly of this new subgenus. Molecular phylogeny based on ML analysis of mtDNA cyt b sequences also separates *Larroussius* from *Lewisius* very clearly. One can of course doubt the relevance of the cyt b marker in obtaining a robust molecular phylogeny including such varied species. Since position of the subgenera *Phlebotomus*, *Paraphlebotomus*, *Artemievus* and *Synphlebotomus* are doubtful, and the apparent paraphyly of *Paraphlebotomus* in the present study contrasts with the robust monophyly obtained on genome-wide data, it was possible to individualize the subgenus *Artemievus* by excluding *Ph. alexandri* of *Paraphlebotomus* [12]. Our goal was not to reconstruct a phylogeny of the genus *Phlebotomus* but to test the monophyly of *Lewisius* as well as its relationships with the *Larroussius*. Moreover, the *Lewisius* has the subgenus *Madaphlebotomus* as sister group. This relationship between Phlebotomine sandflies from Madagascar and from Southeast Asia deserves to be explored in the light of more conserved molecular markers.

Proteomic data, based on MALDI-TOF mass spectrometry, are consistent with the molecular analysis. With this technique, a high distance separates spectra from specimens of *Larroussius* subgenus and those of the subgenus *Lewisius.* Use of MALDI-TOF is also very promising for identification of the two newly described species *Ph. breyi* and *Ph. sinxayarami.* No fresh specimens of *Ph. betisi* were, however, available for MALDI-TOF analysis, and thus did not allow us to fully test the discrimination capabilities of this technique. The constitution of a MALDI-TOF spectral database for Asian sandflies would be particularly interesting for rapid and inexpensive screening.

Morphometric data are coherent with molecular and proteomic approaches. Differences in sizes of wings between male populations are significant. Nonetheless, the morphometric evidence of this current work does not provide an unequivocal result. Principal Component Analysis of the females showed low segregation, whereas males of the two species were more easily separated.

As a side note concerning the biology of this species, it is worth mentioning that our specimen was captured at the entrance of the cave and that we detected a blood meal from a water buffalo in its abdomen. These buffalo were present in a rice field located below the cave at a distance of about 100 to 200 meters from the entrance. They could not access the cave. This trophic preference thus indicates that *Ph. sinxayarami* females have to leave the cave in order to take their blood meals outside, and therefore do not remain permanently inside the cave.

## Supplementary material

The Supplementary materials of this article are available at https://www.parasite-journal.org/10.1051/parasite/2023021/olm.
